# Joint association between physical exercise, caffeine intake, and biological ageing: A cross-sectional analysis of population-based study

**DOI:** 10.1371/journal.pone.0323264

**Published:** 2025-05-07

**Authors:** Guang Chen, Shichen Zhou, Yunqing Xun, Tung Leong Fong, Guoyi Tang, Jingyi Wang, Hongzheng Li, Xiangjun Yin, Jialiang Gao, Guanghui Zhu, Ying Wu, Jinlin Li, Ya Xuan Sun, Yige Li, Jiayan Zhou, Yibin Feng

**Affiliations:** 1 School of Chinese Medicine, Li Ka Shing Faculty of Medicine, The University of Hong Kong, Hong Kong SAR, PR of China; 2 Experimental Research Center, China Academy of Chinese Medical Sciences, Beijing, China; 3 Guang’anmen hospital, China Academy of Chinese Medical Sciences, Beijing, China; 4 School of Basic Medical Science, Zhejiang Chinese Medical University, Hangzhou, China; 5 Harvard Law School, Harvard University, Cambridge, Massachusetts, United States of America; 6 PBC School of Finance, Tsinghua University, Beijing, China; 7 T.H. Chan School of Public Health, Harvard University, Boston, Massachusetts, United States of America; 8 Department of Health Care Policy, Harvard Medical School, Harvard University, Boston, Massachusetts, United States of America; 9 School of Medicine, Stanford University, Stanford, California, United States of America.; Sichuan University, CHINA

## Abstract

**Background:**

Ageing is a significant risk factor for age-related diseases, accounting for 51% of global total disease burden. As thus, promoting healthy ageing is crucial. Although several potential anti-ageing drugs show promise, none have been approved for anti-ageing purpose. The World Health Organization (WHO) recommends physical exercise exceeding 600 metabolic equivalent of task (MET) minutes per week for adults. However, whether physical exercise positively impacts healthy biological ageing remains unclear.

**Objective:**

This study aimed to investigate the joint correlation between MET level, caffeine consumption, and biological ageing.

**Methods:**

We analyzed data from seven survey cycles (2007–2020) of the National Health and Nutrition Examination Survey (NHANES), involving 23,739 participants. Physical activity levels were measured in MET minutes per week, and biological ageing was assessed using both the PhenoAge and ENABL Age algorithms. Generalized linear regression for survey data was employed to test correlations, adjusting for confounding factors. A cubic spline model was used to detect non-linear relationships. Pre-specified subgroup analyses explored effect modifications, while predefined sensitivity analyses confirmed the robustness of the results.

**Results:**

Each 100-MET increase in weekly physical exercise was associated with a 0.2-year delay in biological ageing (p < 0.001 for both PhenoAge and ENABL Age). Individuals with less than 600 MET minutes of weekly exercise had a higher risk of accelerated ageing compared to those exceeding 600 MET minutes (mean difference [MD]: 2.2 PhenoAge years, 95% CI [1.5–2.8], p < 0.001; MD: 2.1 ENABL Age years, 95% CI [1.1–3.1], p < 0.001). A L-shaped association was observed, with diminishing benefits of delayed ageing beyond 292 MET minutes for PhenoAge and 259 MET minutes for ENABL Age. Daily caffeine intake did not modify the correlation between MET levels and biological ageing (p for interaction > 0.05). Stronger effects were observed in non-Hispanic Black individuals, those with obesity, and low-income populations, but no benefit was found in cancer patients.

**Conclusions:**

Our findings highlight a positive correlation between higher levels of weekly physical exercise and delayed biological ageing. However, the benefits plateau beyond specific MET thresholds. Caffeine intake does not influence this relationship. These results underscore the importance of promoting physical exercise at appropriate MET levels as a strategy for healthy ageing management in the general population.

## Introduction

Ageing is defined as the progressive decline in biological function over time [1] and is characterized by 12 key hallmarks, organized into three overarching aspects: (1) causes of damage (genomic instability, telomere attrition, epigenetic alterations, loss of proteostasis, and disabled macroautophagy), (2) responses to damage (deregulated nutrient sensing, mitochondrial dysfunction, and cellular senescence), and (3) culprits of phenotype (stem cell exhaustion, altered intercellular communication, chronic inflammation, and dysbiosis) [2]. An individual’s ageing status can be quantified and estimated using predictive biomarkers identified through phenotype and omics studies [3,4]. These biomarkers are incorporated into algorithms such as DNAmAge [5], GlycanAge [6], GrimAge [7], PhenoAge [8], and ENABL Age [9], enabling the evaluation of longevity interventions [10]. Ageing research is both crucial and unique because ageing is a primary risk factor for a wide range of age-related diseases, including various cancers, neurodegenerative disorders, cardiovascular diseases, and metabolic conditions such as diabetes [11]. Notably, an updated Global Burden of Disease (GBD) study revealed that these age-related diseases account for 51.3% (95% CI: 48.5–53.9) of the global disease burden [12]. Consequently, promoting healthy ageing is essential for enhancing both lifespan and quality of life.

Currently, no anti-ageing intervention has been proven to be both effective and safe. Risk factors including overweight [13], a high dietary inflammation index [14], and negative emotion such as anger [15] have been associated with accelerated ageing, while protective factors including regular sleep patterns are linked to delayed ageing [16]. However, no causal relationship between these factors and ageing has been conclusively established. Beyond these physical, emotional, and behavioral factors, researchers globally are actively exploring pharmacological interventions to mitigate ageing. The hallmarks of ageing are defined by the principle that experimental augmentation of these hallmarks accelerates biological ageing, whereas therapeutic interventions targeting them can decelerate, halt, or even reverse the ageing process [2,11,13–16]. Eight pharmacological compounds targeting these ageing hallmarks have been investigated in human trials, including metformin, oxidized Nicotinamide Adenine Dinucleotide (NAD+) precursors, Target of Rapamycin Complex 1 (TORC1) inhibitors, Glucagon-Like Peptide-1 (GLP-1) receptor agonists, probiotics, senolytics, spermidine, and anti-inflammatories [17]. Additionally, vaccines are being explored for their potential to combat ageing and age-related diseases [18]. Despite these advancements, no anti-ageing drug has yet received approval from the U.S. Food and Drug Administration (FDA), and all potential candidates remain controversial due to uncertainties regarding their efficacy and safety in humans [19].

The World Health Organization (WHO) strongly recommends that adults engage in at least 600 metabolic equivalent of task (MET) minutes per week, which translates to 75 minutes of vigorous-intensity or 150 minutes of moderate-intensity physical activity weekly [20]. Similarly, the Physical Activity Guidelines for Americans advocate for a minimum of 600 MET minutes per week, supplemented by muscle-strengthening activities on two or more days per week [21]. Despite these recommendations, the relationship between MET levels in physical exercise and healthy ageing remains unclear, particularly in subpopulations with chronic diseases such as cancer survivors. While higher physical activity levels following cancer diagnosis and treatment have been associated with reduced mortality in breast, prostate, gynecological, and colorectal cancers, this correlation is less certain in lung cancer patients [22]. Additionally, many cancer survivors face challenges in performing and adhering to regular physical exercise due to fluctuating cancer-related symptoms and demanding treatment schedules [23].

In parallel with physical activity, caffeine consumption has gained attention for its potential influence on biological ageing. Research indicates that caffeine intake is associated with improved cognitive performance, a recognized phenotype of biological ageing [24]. Additionally, a randomized, double-blind, crossover, placebo-controlled study among cyclists demonstrated that caffeine enhances exercise performance and provides cardio-protective effects during intense physical activity [25]. Despite these promising findings, the combined impact of exercise and caffeine consumption on biological ageing remains underexplored. Therefore, our study aimed to investigate the joint correlation between MET levels, caffeine consumption, and biological ageing.

## Materials and methods

### Study design and population

The National Health and Nutrition Examination Survey (NHANES), a nationwide cross-sectional survey, investigates the nutrition and health conditions in the US population (https://www.cdc.gov/nchs/nhanes/index.htm). This study was approved by the national center for health statistics ethics review board (Approval number: #2018-01). All participants provided the written consent before the enrollment, and the written consent can be found on the NHANES website (https://wwwn.cdc.gov/nchs/nhanes/continuousnhanes/documents.aspx?BeginYear=2017). The information collected in this survey encompassed demographics, socioeconomic status, lifestyle and health questionnaires, and bio-specimen data. In this study, we included seven NHANES cycles (2007–2008, 2009–2010, 2011–2012, 2013–2014, 2015–2016, 2017–2018, 2019–2020). Participants were excluded if they were 18 years of age or younger, pregnant, or had missing data on physical exercise.

### Measurement of exposure

The participant’s weekly physical exercise and activity amount was measured by MET in the NHANES database. MET per week was calculated by vigorous work and recreational activity minutes per week, moderate work and recreational activity minutes per week, and walk or bicycle minutes per week. Typically, 600 MET is equivalent to 75 minutes of vigorous intensity physical activity or 150 minutes of moderate physical activity per week [20]. Physical exercise was categorized into two classes based on the WHO recommendation for adults: adequate level of physical exercise (MET ≥ 600 per week) and inadequate level of physical exercise (MET < 600 per week) [20]. Daily caffeine consumption primarily originates from coffee, tea, cola drinks, and chocolate. Participants’ caffeine consumption was quantified in urine (umol/L) using high-performance liquid chromatography-electrospray ionization-tandem quadrupole mass spectrometry in the HNANES database [24].

### Ascertainment of outcomes

Biological ageing was calculated for both PhenoAge [8] and ENABL Age [9] for all participants surveyed in NHANES. PhenoAge was calculated based on chronological age and clinical biomarkers which correspond to estimated mortality risk, including concentration of albumin, glucose, C-reactive protein (CRP), alkaline phosphatase, creatinine, red blood cell distribution width, mean cell volume, lymphocyte percent, and white blood cell count. ENABL Age, also incorporating clinical biomarkers, can distinguish unhealthy ageing from the healthy ones, and predict 5-year mortality with the power of an area under the receiver operating characteristic (ROC) curve of 0.89 and 0.91 for 10-year mortality on the NHANES dataset.

### Extraction of confounding factors

The factors that were unbalanced across exercise exposure groups and were shown to be correlated with ageing, were defined as confounding factors in this study [26]. In our study, confounding factors include sex (female/male), race (Non-Hispanic Black/Non-Hispanic White/Other Hispanic/ Mexican American/other race including multi-racial), body mass index (BMI) (≤25 kg/m^2^ as normal, > 25 to < 30 kg/m^2^ as overweight, ≥ 30 kg/m^2^ as obesity), marital status (married/divorced/widowed/separated/never married/living with partner), income (assessed by income poverty ratio), sleep disorder (with/without), smoking status (never or ever), alcohol intake (never or ever), and history of cancer diagnosis (never or ever) [26].

### Statistical analysis

Baseline characteristics were calculated and listed across groups with MET < 600 and MET ≥ 600 using Python package *tableone*. Continuous variables were described in mean (SD) or median (IQR); categorical variables were shown as absolute numbers along with percentages. According to the statistical guidance on the NHANES website, modeling weights were added based on sampling design and weights for each survey cycle in our analysis model s*vyglm* in R. All regression analyses were controlled for confounding factors by adding covariates in the multivariate regression models.

We used generalized linear regression model to test the correlation between total MET per week and biological ageing, between MET category (< 600 or ≥ 600) and biological ageing, controlled for confounding factors including race, sex, income, marital status, BMI, sleep disorder, alcohol intake, smoking, and history of cancer, while incorporating the NHANES weights across each survey cycle.

Non-linear correlation was explored by the cubic spline models between total MET per week and biological ageing. The non-linear *P* value indicates whether there was statistically non-linear association, and the cut-off point was detected if non-linear correlation existed.

Subgroup analysis was conducted to figure out effect modifiers, where we stratified the regression models by sex (male vs. female), race (Non-Hispanic Black vs. Non-Hispanic White vs. Other Hispanic vs. Mexican American vs. other race), BMI (≤25 vs. > 25 to < 30 vs. ≥ 30 kg/m^2^), family income poverty ratio (1 vs. 1–4 vs. > 4), smoking status (current smoker vs. former smoker vs. nonsmoker), and history of cancer diagnosis (with vs. without) [26].

The following sensitivity analyses were also conducted to test the robustness of the regression results. First, we excluded participants with cancer because cancer is shown to affect the exercise ability and also negatively affect the healthy ageing. Second, we did the sensitivity analyses to examine the association between biological ageing and weekly vigorous intensity physical activity. Third, we did the sensitivity analyses to examine the association between biological ageing and daily vigorous intensity and moderate intensity physical activity.

We used R package *rcssci* for cubic spline models and verified the results by *porstrcspline* in *Stata* package. All other analysis and plot were conducted using R, with statistical significance set at a p-value of 0.05.

## Results

### Baseline characteristics and descriptive statistics

**Fig 1:**
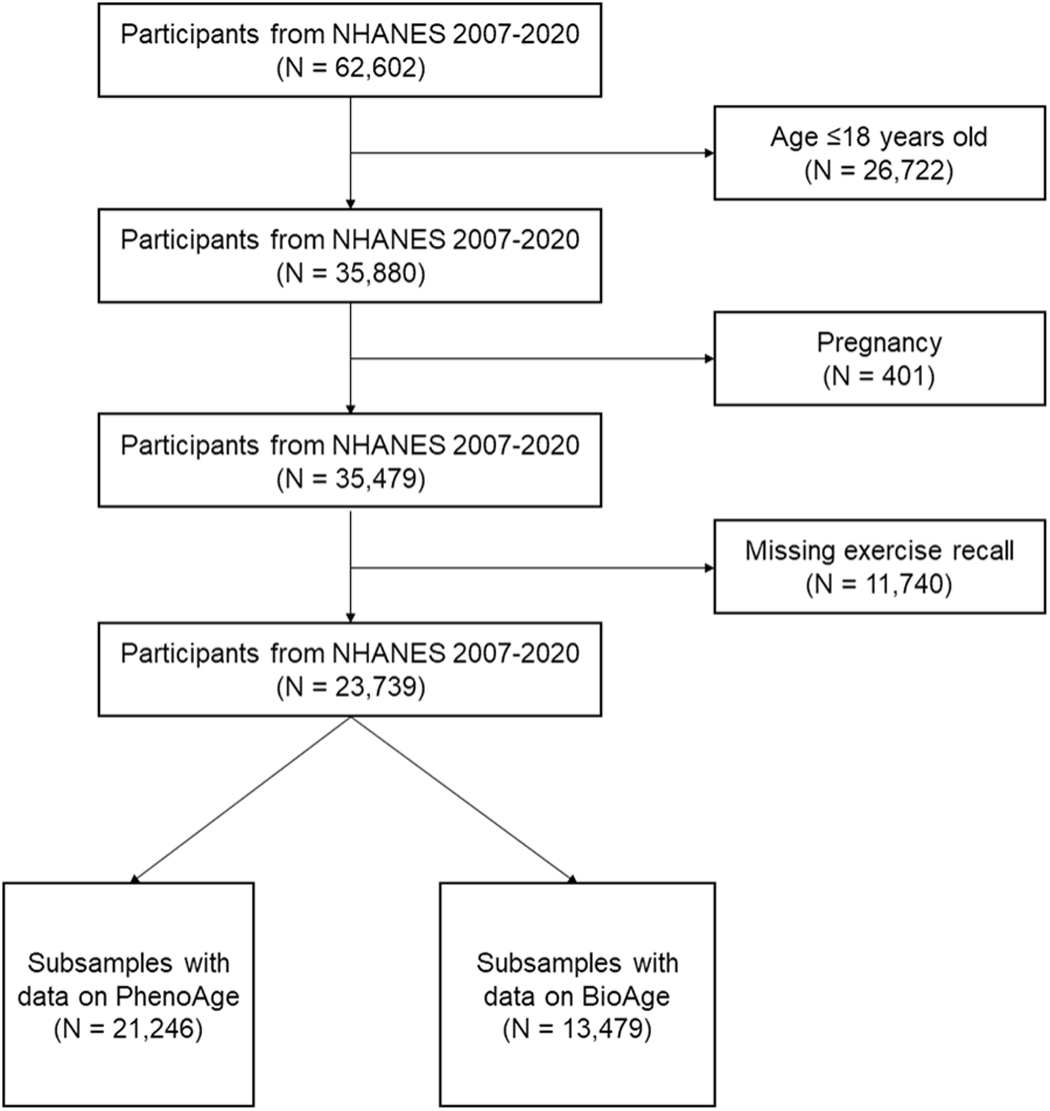
Flow chart for participant selection in NHANES. NHANES, National Health and Nutrition Examination Survey.

Out of 62,602 participants enrolled in the NHANES study from 2007 to 2020, a total of 23,739 were included in the final analysis (Fig 1). During participant selection, data on exercise were missing for 11,740 participants, representing 19% of the total cohort. Table 1 provided a summary of the baseline characteristics of the included participants stratified by cancer status. Participants included in this study has a mean age of 46.1 years (SD = 17.5), a mean BMI of 28.7 kg/m^2^ (SD = 6.7) with 52.1% of female. Significant differences in baseline characteristics were observed across groups, shown in Table 1.

**Table 1:** Baseline characteristics of included participants across exercise intensity based on MET.

Characteristics	Overall (N = 23739)	MET < 600 (N = 14250)	MET ≥ 600 (N = 9489)	P-Value
**Age (years)**	46.1 (17.5)	47.8 (17.6)	43.6 (17.0)	<0.001^*^
**Gender %**				<0.001^*^
**Female**	12362 (52.1)	6713 (47.1)	5649 (59.5)	
**Male**	11377 (47.9)	7537 (52.9)	3840 (40.5)	
**Race %**				<0.001^*^
**Mexican American**	3335 (14.0)	1840 (12.9)	1495 (15.8)	
**Other Hispanic**	2418 (10.2)	1358 (9.5)	1060 (11.2)	
**Non-Hispanic White**	9683 (40.8)	5771 (40.5)	3912 (41.2)	
**Non-Hispanic Black**	5086 (21.4)	3009 (21.1)	2077 (21.9)	
**Other Race**	3217 (13.6)	2272 (15.9)	945 (10.0)	
**Marital status %**				<0.001^*^
**Married**	12293 (53.5)	7640 (55.1)	4653 (51.1)	
**Widowed**	1981 (8.6)	1323 (9.5)	658 (7.2)	
**Divorced**	2972 (12.9)	1774 (12.8)	1198 (13.1)	
**Separated**	538 (2.3)	312 (2.2)	226 (2.5)	
**Never married**	3742 (16.3)	2040 (14.7)	1702 (18.7)	
**Living with partner**	1442 (6.3)	775 (5.6)	667 (7.3)	
**Income poverty ratio**	2.6 (1.7)	2.8 (1.7)	2.4 (1.6)	<0.001^*^
**Body Mass Index (kg/m2)**	28.7 (6.7)	28.6 (6.7)	28.9 (6.7)	0.010^*^
**Sleep disorders %**				0.006^*^
**Yes**	5506 (23.2)	3387 (23.8)	2119 (22.3)	
**No**	18227 (76.8)	10859 (76.2)	7368 (77.6)	
**Smoking status %**				<0.001^*^
**Yes**	4455 (45.8)	2267 (41.3)	2188 (51.7)	
**No**	5263 (54.1)	3217 (58.7)	2046 (48.3)	
**Drinking status %**				<0.001^*^
**Yes**	16115 (78.4)	9417 (76.7)	6698 (81.0)	
**No**	4424 (21.5)	2857 (23.3)	1567 (19.0)	
**Self-reported cancer %**	1892 (8.2)	1256 (9.1)	636 (7.0)	<0.001^*^

Variables are presented as means ± standard deviation or count with percentage. MET, metabolic equivalent of task.

*Represents significant differences between groups using an independent sample t-test or Chi-squared test. Alpha level at 0.05.

### Association of MET with biological ageing

The main associations between biological ageing and physical exercise were analyzed using both continuous variables (total MET per week) and binary variables (inadequate physical exercise: MET < 600 vs. adequate physical exercise: MET ≥ 600). These associations were assessed in both unadjusted linear regression models and models adjusted for covariates, as presented in Table 2. Individual with less than 600-MET physical exercise per week had a higher risk of ageing acceleration than those who had more than 600-MET physical exercise per week (MD 2.2 PhenoAge years, 95% CI [1.5–2.8], p < 0.001; MD 2.1 ENABL Age years, 95% CI [1.1–3.1], p < 0.001). In particular, each 100-MET increase per week in physical exercise was associated with delayed biological ageing by 0.2 PhenoAge years (p < 0.001), adjusted for all the measured confounding factors including sex, race, marital status, BMI, income, alcohol intake, smoking status, sleep disorder, and history of cancer, whereas each 100-MET increase in physical exercise per week was correlated with attenuated biological ageing by 0.2 ENABL Age years (p < 0.001), adjusted for the same confounding factors.

**Table 2:** Associations between MET per week with biological ageing.

Biological ageing	Model I β(95% CI)	P value	Model II β(95% CI)	P value	Model III β(95% CI)	P value
**PhenoAge (years)**	–0.3 (–0.3, –0.2)	<0.001*	–0.2 (–0.3, –0.2)	<0.001*	–0.2 (–0.2, –0.1)	<0.001^*^
** MET < 600**	Reference		Reference		Reference	
** MET ≥ 600**	–3.0 (–3.7, –2.3)	<0.001*	–2.4 (–3.1, –1.6)	<0.001*	–2.2 (–2.8, –1.5)	<0.001^*^
**ENABL Age (years)**	–0.3 (–0.3, –0.2)	<0.001*	–0.2 (–0.3, –0.1)	<0.001*	–0.2 (–0.3, –0.1)	<0.001^*^
** MET < 600**	Reference		Reference		Reference	
** MET ≥ 600**	–2.9 (–3.7, –2.1)	<0.001*	–2.0 (–2.8, –1.2)	<0.001*	–2.1 (–3.1, –1.1)	<0.001^*^

Results were presented in point estimate and 95% Confidence interval. Model I: raw model without covariates to adjust; Model II: adjusted for gender, race, marital status, income; Model III: adjusted for covariates in model II and BMI, sleep disorder, smoking, alcohol intake, history of cancer. The independent variable unit is per 100-MET change in all models. MET, metabolic equivalent of task.

*Represents significant differences between groups using generalized linear regression model. Alpha level at 0.05.

**Fig 2:**
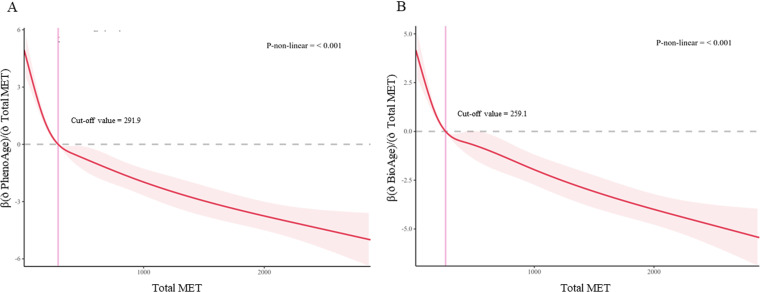
Nonlinear associations between MET and biological age based on restricted cubic spline models. The cubic spline models also adjusted for gender, race, BMI, sleep disorder, smoking, alcohol intake, and history of cancer. The solid blue line represents the smooth curve fit between dependent and independent variables. The grew bands represent the 95% confidence interval from the fit. **(A)** PhenoAge; **(B)** ENABL Age.

In the nonlinear association analysis, adjusted cubic spline model demonstrated a non-linear association between the total MET level and biological ageing (non-linear p < 0.001 for both PhenoAge and ENABL Age). A L-shaped association was observed in which the benefit of delayed ageing got weaker when individual’s physical exercise level exceeds the cut-off of 292-MET for PhenoAge or exceeds the cut-off of 259-MET for ENABL Age, respectively (Fig 2), where these cut-off points were calculated and generated from the cubic spline models adjusted for confounding factors.

### Joint analysis of physical exercise and caffeine consumption on biological ageing

**Fig 3:**
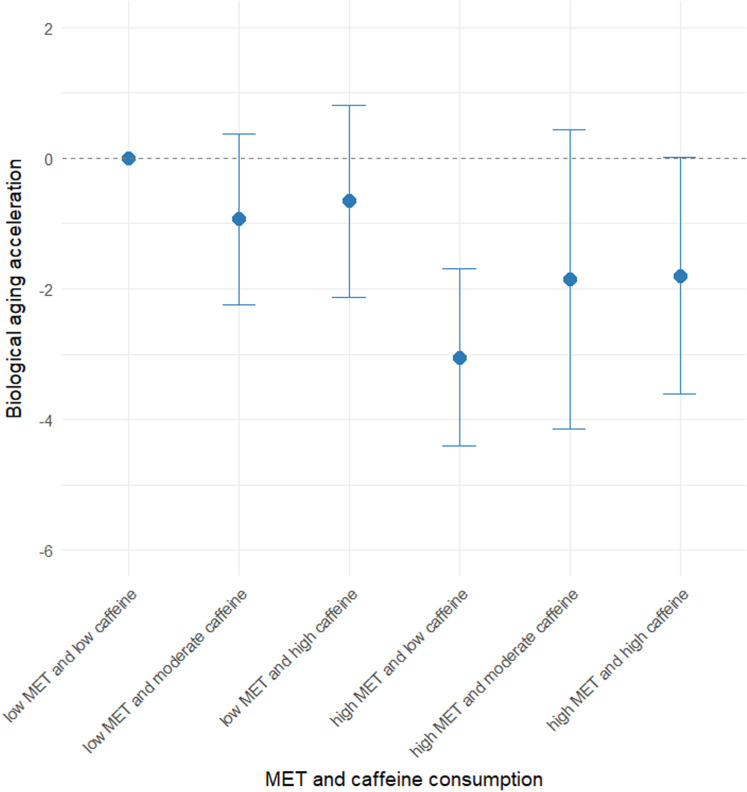
Joint association of exercise and caffeine consumption with biological ageing acceleration. MET is categorized as low and high by the cutoff of 600 MET per week. Caffeine consumption is categorized as low, moderate, and high by the cutoff of 100 and 200 umol/L.

The joint associations between physical exercise and caffeine consumption was illustrated in Fig 3. We found that combination of high MET and lower caffeine consumption were associated with delayed biological ageing (MD -3.05, 95% CI = -4.41 to -1.69). Conversely, combinations of moderate and high levels of caffeine consumption attenuated the reductions in biological ageing of physical exercise, such as combination of > 600 MET and > 200 umol/L caffeine consumption (p for interaction = 0.886).

### Stratified subgroup analysis

**Fig 4:**
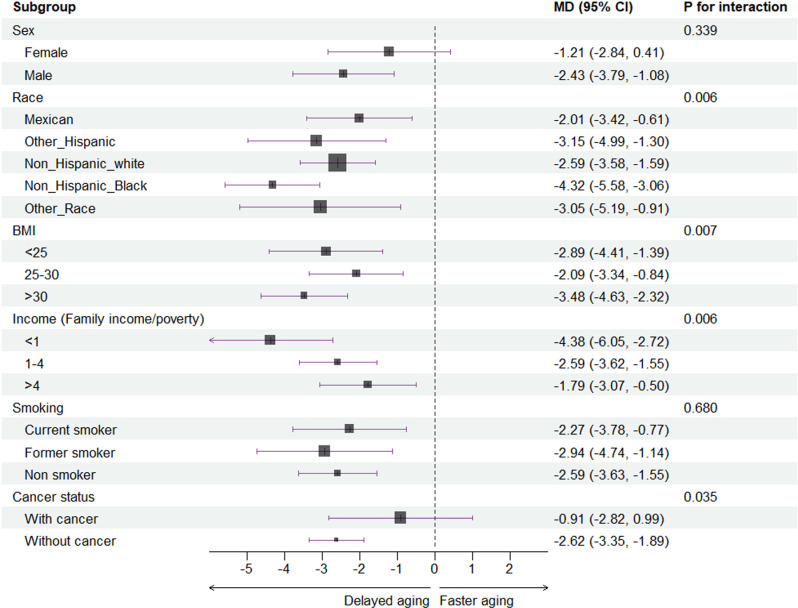
Subgroup analyses of associations between MET and biological age by effect modifiers of sex, race, BMI, income, sleep disorder, smoking, and self-reported cancer.

The association between physical exercise level and biological age were stronger in non-Hispanic Black (MD = -4.32, 95% CI: -5.58 to -3.06) than in other races (p for interaction = 0.006) and in participants with obesity (MD = -3.48, 95% CI: -4.63 to -2.32) than in those with overweight or normal BMI (p for interaction = 0.007), which were shown in Fig 4. The magnitude of benefit observed in low-income participants (family income poverty ratio less than 1) was significantly larger (MD = -4.38, 95% CI: -6.05 to -2.72) than middle-class or rich participants (p for interaction = 0.006). Stratification analysis by history of cancer diagnosis revealed significantly different results (p for interaction = 0.035) between participants with and without cancer. The findings indicated that cancer patients did not experience significant benefits from increased physical exercise in terms of delayed ageing (MD = -0.91, 95% CI: -2.82 to 0.99). The interaction of other stratified analyses was not significant.

### Sensitivity analysis

The pre-specified sensitivity analyses did not change the observed results. A similar pattern of associations between physical exercise level and delayed ageing was observed in all sensitivity analyses, including regression models after excluding cancer patients, association analyses using various physical exercise level measurement approaches (MET per week, vigorous intensity activity minutes per week, vigorous intensity activity minutes per day), and using different physical exercise level category ascertainment approaches (MET ≥ 600 per week, vigorous intensity activity ≥ 75 minutes per week, vigorous intensity activity ≥ 30 minutes per day) (S1 Table and S2 Table).

## Discussion

In our study, we conducted a cross-sectional analysis to explore the correlation between physical activity levels (measured in MET) and biological ageing using data from the NHANES study. Our findings revealed that higher MET levels were associated with delayed biological ageing in adults without cancer. Specifically, participants who engaged in more than 600 MET of exercise per week exhibited delayed biological ageing by an average of 2.2 years compared to those with less than 600 MET per week. Notably, stronger benefits of biological ageing deceleration were observed among individuals of non-Hispanic Black ethnicity, those with obesity, and those with lower income levels. Furthermore, our results suggest that cancer patients might not experience significant benefits from higher levels of physical exercise in terms of biological ageing.

The results of this study align with previous research indicating that exercise can decelerate the ageing process through various mechanisms. In mouse models, running has been shown to reduce leptin production in adipose tissue, decrease hematopoietic activity, and subsequently lower chronic inflammation [27]. Another study in mice demonstrated that exercise not only mitigated the upregulation of inflammatory pathways in older mice but also restored intercellular communication within stem cell compartments through immune cells [28]. A pilot study involving 45 participants found that moderate-to-vigorous physical activity attenuated the premature senescence of immune cells [29]. A population-based study revealed that different exercise patterns were associated with ageing outcomes, such as the correlation of leisure walking with delayed ageing and job-related physical activities with accelerated ageing [30]. Additionally, a cohort study in Germany found that MET levels were linked to ageing-related epigenetic features, as measured by DNA methylation sequencing [31].

### Non-linear correlation interpretation

The non-linear correlation between physical exercise levels and biological ageing may be explained by recent findings suggesting that vigorous exercise can increase biomarkers of cardiomyocyte injury, indicating that lifelong endurance exercise may contribute to myocardial scarring [32]. This raises the possibility that higher levels of physical exercise (e.g., exceeding 292 MET for PhenoAge or 259 MET for ENABL Age) may induce other forms of bodily damage, potentially offsetting the beneficial effects of exercise on biological ageing. For instance, in the context of neuro-ageing, a cross-sectional study found that high-intensity physical activity was not associated with improved cognitive performance in older adults [33]. Our findings regarding the non-linear relationship suggest that the beneficial effects of exercise may vary across different activity intensities, highlighting the need for a balanced approach to physical activity.

### Effect modification interpretation

The differential benefits of physical exercise on biological ageing among non-Hispanic Black individuals, those with obesity, and low-income populations, as well as the lack of benefit observed in cancer patients, can be attributed to several factors. Non-Hispanic Black individuals experience greater delays in biological ageing compared to other racial groups, which may be partially explained by a metabolic profiling study showing that metabolite responses to physical activity are dose-sensitive and vary by race, with Black populations exhibiting a more pronounced response than White populations [34]. For individuals with obesity, a Mendelian randomization study demonstrated a causal link between overweight status and accelerated biological ageing [35], suggesting that physical exercise may decelerate ageing through weight loss as a mediating factor. Regarding socioeconomic status, a secondary analysis of the NHANES study revealed a positive correlation between higher income and greater physical activity levels in adolescents and young adults [36], while a longitudinal U.S. study from 2001 to 2014 found that higher income was associated with increased longevity [37]. The economic principle of diminishing marginal utility may explain why low-income individuals, despite having fewer resources for exercise facilities, experience greater benefits in terms of delayed biological ageing. This reflects the idea that providing additional benefits to those with fewer resources (e.g., low-income individuals) yields greater overall impact compared to those who already have ample resources (e.g., high-income individuals) [38,39].

### Cancer population interpretation

Although an analysis of the Global Burden of Disease (GBD) study found that physical exercise is inversely correlated with the risk of breast, lung, gastric, liver, and colon cancers [40], this does not necessarily imply that individuals diagnosed with cancer can still benefit from physical exercise, particularly those experiencing cancer-related fatigue following surgery, chemotherapy, or other treatments. The overlapping hallmarks of ageing and cancer provide valuable insights into understanding the complex relationship between exercise levels and biological ageing in cancer patients [41]. Among the 12 hallmarks of ageing, genomic instability, epigenetic alterations, chronic inflammation, and dysbiosis share commonalities with cancer, exerting similar directional effects. However, telomere attrition and stem cell exhaustion, which accelerate ageing, paradoxically suppress oncogenesis. Additionally, disabled macro-autophagy and cellular senescence, which promote ageing, exhibit scenario-dependent effects that can either support or inhibit tumorigenesis. The intricate interplay of these hallmarks partly explains the complicated relationship between physical exercise and biological ageing in cancer patients.

### Interaction with caffeine

Joint analysis revealed that caffeine consumption did not modify the correlation between physical exercise and biological ageing, as no interaction between caffeine consumption and physical exercise was observed—contrary to our initial assumption. A previous epidemiological study using NHANES data found that caffeine intake was inversely associated with telomere length, while coffee consumption was positively correlated with telomere length [42]. This discrepancy may explain why moderate and high levels of caffeine consumption attenuated the positive correlation between physical exercise and biological ageing. The underlying mechanism of physical exercise’s effect on biological ageing is related to mechanotransduction, a process by which organisms convert mechanical loading into cellular responses [43]. In contrast, caffeine, a 1,3,7-trimethylxanthine, functions as a non-selective adenosine receptor antagonist, affecting multiple systems in the body [44]. Although previous research has demonstrated that caffeine intake can enhance physical exercise performance [45], our study did not observe any interaction between caffeine consumption and physical exercise in terms of biological ageing.

What have been reported regarding the relationship between physical exercise, caffeine intake, and biological ageing were summarized in Table 3.

**Table 3:** Summary of studies investigating association of exercise, caffeine, and ageing.

Study system	Exposure	Outcome	Key findings
Mice model [27]	Running	Ageing-related biomarkers	Running diminished leptin production in adipose tissue, decrease hematopoietic activity, and reduced chronic inflammation
Mice model [28]	Exercise	Ageing-related pathways	Exercise ameliorated the upregulation of inflammatory pathways in old mice
Pilot study on human [29]	Moderated-vigorous physical activity	Premature senescence of immune cells	Moderated-vigorous physical activity attenuated the premature senescence of immune cells in 45 participants
Cross-sectional study on human [30]	Physical activity patterns	Leukocyte telomere length and biological age acceleration	Physical activity, such as strenuous sports and other exercises in leisure time and the use of public transportation, was associated with reduced biological ageing
Cohort study on human [31]	MET level	Ageing-related epigenetic features	Regular physical activity slowed epigenetic ageing by counteracting immunosenescence and lowering cardiovascular risk
Cross-sectional study on human [33]	High intensity physical activity	Cognitive performance	High intensity physical activity was not related to improved cognitive performance in the elderly
Cross-sectional study on human [42]	Caffeine intake	Telomere length	Caffeine intake was inversely associated with telomere length

RCT, randomized controlled trial; MET, metabolic equivalent of task

### Strengths and limitations

Strengths of this study include the following points. First, we utilized the NHANES dataset, which features a large sample size and long survey periods, providing sufficient statistical power for our analyses. NHANES’s weighted sampling methods ensure that our study sample is representative of the entire U.S. population. Second, we conducted subgroup analyses to explore potential effect modifiers and sensitivity analyses to confirm the consistency of our findings, thereby enhancing the robustness of our results and conclusions. This study also had the following limitations. First, due to the nature of cross-sectional studies, we cannot establish causal relationships between physical exercise and delayed biological ageing. The observed non-linear associations are exploratory and require further validation. Second, although we controlled for potential confounding factors as covariates in our regression models, residual and unmeasured confounding factors might still bias our results. Third, phenotype and biomarker data for biological ageing were only available at one time point, limiting our ability to capture and investigate changes in biological ageing over time. Forth, physical activity levels were calculated based on self-reported questionnaires about daily or weekly habits, which may introduce information and measurement errors, potentially biasing our results. Finally, the finding that more than 600 METs of physical exercise per week was associated with an average of 2.2 years of delayed biological ageing should be interpreted cautiously. The effects of physical activity on biological ageing are likely not immediate but may manifest as aftereffects or long-term outcomes.

## Conclusions

In conclusion, our findings indicate a positive correlation between higher levels of physical exercise per week and delayed biological ageing among U.S. adults without cancer. However, the benefits of delayed ageing diminish at higher MET levels of physical exercise, and caffeine intake does not modify this correlation. Physical exercise exceeding 600 METs per week—equivalent to 75 minutes of vigorous-intensity or 150 minutes of moderate-intensity activity per week—may be particularly advantageous in decelerating biological ageing as part of healthy ageing management. These findings are constrained by the limitations of the cross-sectional design and the inability to establish causal relationships. Nevertheless, the results hold significant implications for public health strategies aimed at promoting healthy ageing through appropriate levels of physical activity. Further validation using prospective cohort studies or interventional trials is warranted to confirm these observations.

## Supporting information

Checklist STROBE statement. An Explanation and Elaboration article discusses each checklist item and gives methodological background and published examples of transparent reporting. The STROBE checklist is best used in conjunction with this article (freely available on the Web sites of PLoS Medicine at http://www.plosmedicine.org/, Annals of Internal Medicine at http://www.annals.org/, and Epidemiology at http://www.epidem.com/). Information on the STROBE Initiative is available at www.strobe-statement.org.
(DOCX)

S1 Table Sensitivity analyses of associations between MET and biological age excluding participants with cancer. Model I: raw model without covariates to adjust; Model II: adjusted for gender, race, marital status, income; Model III: adjusted for covariates in model II and BMI, sleep disorder, smoking, alcohol intake, history of cancer. The independent variable unit is per 100-MET change in all models. MET, metabolic equivalent of task.
(DOCX)

S2 Table Sensitivity analyses of associations between physical activity with biological ageing. Model I: raw model without covariates to adjust; Model II: adjusted for gender, race, marital status, income; Model III: adjusted for covariates in model II and BMI, sleep disorder, smoking, alcohol intake, history of cancer. MET, metabolic equivalent of task. Vigorous activities per day > 75 minutes or moderate activities per day > 150 minutes as high intensity; low intensity otherwise.
(DOCX)
