# Supplementary material for: Joint association between physical exercise, caffeine intake, and biological ageing: A cross-sectional analysis of population-based study
Source: PLoS One. 2025 May 7;20(5):e0323264. doi: 10.1371/journal.pone.0323264 (PMC12058179; doi:10.1371/journal.pone.0323264)
Supplement: S1 Table — Model I: raw model without covariates to adjust; Model II: adjusted for gender, race, marital status, income; Model III: adjusted for covariates in model II and BMI, sleep disorder, smoking, alcohol intake, history of cancer. The independent variable unit is per 100-MET change in all models. MET, metabolic equivalent of task. (DOCX) [file pone.0323264.s002.docx]

**S1 Table. Sensitivity analyses of associations between MET and biological age excluding participants with cancer.**

| Biological ageing | Model I  β(95% CI) | P value | Model II  β(95% CI) | P value | Model III  β(95% CI) | P value |
| --- | --- | --- | --- | --- | --- | --- |
| PhenoAge (years) | -0.2 (-0.3, -0.2) | < 0.001* | -0.2 (-0.2, -0.1) | < 0.001* | -0.2 (-0.2, -0.1) | < 0.001* |
| MET < 600 | Reference |  | Reference |  | Reference |  |
| MET ≥ 600 | -2.5 (-3.2, -1.9) | < 0.001* | -2.1 (-2.8, -1.4) | < 0.001* | -2.1 (-2.8, -1.4) | < 0.001* |
| ENABL Age (years) | -0.2 (-0.3, -0.2) | < 0.001* | -0.2 (-0.2, -0.1) | < 0.001* | -0.3 (-0.4, -0.1) | < 0.001* |
| MET < 600 | Reference |  | Reference |  | Reference |  |
| MET ≥ 600 | -2.6 (-3.3, -1.9) | < 0.001* | -1.9 (-2.7, -1.2) | < 0.001* | -2.5 (-4.4, -0.8) | 0.006* |

Results were presented in point estimate and 95% Confidence interval. Model I: raw model without covariates to adjust; Model II: adjusted for gender, race, marital status, income; Model III: adjusted for covariates in model II and BMI, sleep disorder, smoking, alcohol intake, history of cancer. The independent variable unit is per 100-MET change in all models. MET, metabolic equivalent of task

* Represents significant differences between groups using generalized linear regression model. Alpha level at 0.05.
