# Supplementary material for: Joint association between physical exercise, caffeine intake, and biological ageing: A cross-sectional analysis of population-based study
Source: PLoS One. 2025 May 7;20(5):e0323264. doi: 10.1371/journal.pone.0323264 (PMC12058179; doi:10.1371/journal.pone.0323264)
Supplement: S2 Table — Model I: raw model without covariates to adjust; Model II: adjusted for gender, race, marital status, income; Model III: adjusted for covariates in model II and BMI, sleep disorder, smoking, alcohol intake, history of cancer. MET, metabolic equivalent of task. Vigorous activities per day > 75 minutes or moderate activities per day > 150 minutes as high intensity; low intensity otherwise. (DOCX) [file pone.0323264.s003.docx]

**S2 Table. Sensitivity analyses of associations between physical activity with biological ageing**

| Biological ageing | Model I  β (95% CI) | P value | Model II  β (95% CI) | P value | Model III  β (95% CI) | P value |
| --- | --- | --- | --- | --- | --- | --- |
| PhenoAge (years) |  |  |  |  |  |  |
| MET per week | -0.3 (-0.3, -0.2) | <0.001* | -0.2 (-0.3, -0.2) | <0.001* | -0.2 (-0.2, -0.1) | <0.001* |
| Vigorous intensity activity minutes per week | -0.02 (-0.03, -0.02) | <0.001* | -0.02 (-0.03, -0.02) | <0.001* | -0.02 (-0.03, -0.02) | <0.001* |
| Vigorous intensity activity minutes per day | -0.10 (-0.11, -0.09) | <0.001* | -0.09 (-0.10, -0.08) | <0.001* | -0.09 (-0.11, -0.06) | <0.001* |
| MET < 600 per week | Reference |  | Reference |  | Reference |  |
| MET ≥ 600 per week | -3.0 (-3.7, -2.3) | <0.001* | -2.4 (-3.1, -1.6) | <0.001* | -2.2 (-2.8, -1.5) | <0.001* |
| Vigorous intensity activity < 75 mins per week | Reference |  | Reference |  | Reference |  |
| Vigorous intensity activity ≥ 75 mins per week | -9.3 (-10.1, -8.5) | <0.001* | -9.1 (-9.9, -8.3) | <0.001* | -8.4 (-9.9, -7.0) | <0.001* |
| Vigorous intensity activity < 30 mins per day | Reference |  | Reference |  | Reference |  |
| Vigorous intensity activity ≥ 30 mins per day | -9.5 (-10.3, -8.7) | <0.001* | -9.2 (-10.0, -8.3) | <0.001* | -8.9 (-10.7, -7.3) | <0.001* |
| ENABL Age (years) |  |  |  |  |  |  |
| MET per week | -0.3 (-0.3, -0.2) | <0.001* | -0.2 (-0.3, -0.1) | <0.001* | -0.2 (-0.3, -0.1) | <0.001* |
| Vigorous intensity activity minutes per week | -0.02 (-0.02, -0.02) | <0.001* | -0.02 (-0.02, -0.02) | <0.001* | -0.02 (-0.02, -0.01) | <0.001* |
| Vigorous intensity activity minutes per day | -0.08 (-0.09, -0.07) | <0.001* | -0.08 (-0.09, -0.06) | <0.001* | -0.06 (-0.09, -0.04) | <0.001* |
| MET < 600 per week | Reference |  | Reference |  | Reference |  |
| MET ≥ 600 per week | -2.9 (-3.7, -2.1) | <0.001* | -2.0 (-2.8, -1.2) | <0.001* | -2.1 (-3.1, -1.1) | <0.001* |
| Vigorous intensity activity < 75 mins per week | Reference |  | Reference |  | Reference |  |
| Vigorous intensity activity ≥ 75 mins per week | -8.0 (-9.0, -7.1) | <0.001* | -7.8 (-8.8, -6.9) | <0.001* | -6.9 (-8.5, -5.4) | <0.001* |
| Vigorous intensity activity < 30 mins per day | Reference |  | Reference |  | Reference |  |
| Vigorous intensity activity ≥ 30 mins per day | -8.3 (-9.2, -7.4) | <0.001* | -7.9 (-8.9, -6.9) | <0.001* | -7.7 (-9.4, -6.0) | <0.001* |

Results were presented in point estimate and 95% Confidence interval. Model I: raw model without covariates to adjust; Model II: adjusted for gender, race, marital status, income; Model III: adjusted for covariates in model II and BMI, sleep disorder, smoking, alcohol intake, history of cancer. MET, metabolic equivalent of task. Vigorous activities per day > 75 minutes or moderate activities per day > 150 minutes as high intensity; low intensity otherwise.

* Represents significant differences between groups using generalized linear regression model. Alpha level at 0.05.
